# Impaired non‐canonical transforming growth factor‐β signalling prevents profibrotic phenotypes in cultured peptidylarginine deiminase 4‐deficient murine cardiac fibroblasts

**DOI:** 10.1111/jcmm.16915

**Published:** 2021-09-14

**Authors:** Hanane Akboua, Kaveh Eghbalzadeh, Ugur Keser, Thorsten Wahlers, Adnana Paunel‐Görgülü

**Affiliations:** ^1^ Department of Cardiothoracic Surgery Heart Center of the University of Cologne Cologne Germany

**Keywords:** cardiac fibroblasts, fibrosis, PAD4, TGF‐β, transdifferentiation

## Abstract

Transforming growth factor‐β (TGF‐β) becomes rapidly activated in the infarcted heart. Hence, TGF‐β‐mediated persistent activation of cardiac fibroblasts (CFs) and exaggerated fibrotic responses may result in adverse cardiac remodelling and heart failure. Additionally, peptidylarginine deiminase 4 (PAD4) was described to be implicated in organ fibrosis. Here, we investigated the impact of PAD4 on CF function and myofibroblast transdifferentiation *in vitro*. The expression of fibrosis‐related genes was largely similar in cultured WT and PAD4^‐/‐^ CFs of passage 3, although collagen III was reduced in PAD4^‐/‐^ CFs. Exposure to TGF‐β inhibited proliferation and increased contractile activity and migration of WT CFs, but not of PAD4^‐/‐^ CFs. However, under baseline conditions, PAD4^−/−^ CFs showed comparable functional characteristics as TGF‐β‐stimulated WT CFs. Although the SMAD‐dependent TGF‐β pathway was not disturbed in PAD4^‐/‐^ CFs, TGF‐β failed to activate protein kinase B (Akt) and signal transducer and activator of transcription 3 (STAT3) in these cells. Similar results were obtained in WT CFs treated with the PAD4 inhibitor Cl‐amidine. Abrogated Akt activation was associated with diminished levels of phosphorylated, inactive glycogen synthase kinase‐3β (GSK‐3β). Consequently, PAD4^‐/‐^ CFs did not upregulate collagen I and α‐smooth muscle actin (α‐SMA) expression after TGF‐β treatment. Thus, PAD4 is substantially involved in the regulation of non‐canonical TGF‐β signalling and may represent a therapeutic target for the treatment of adverse cardiac remodelling.

## 1 INTRODUCTION

Ischaemic heart disease (IHD) and its consequences are major underlying pathogenic factors of heart failure and contribute significantly to morbidity and mortality worldwide.^1^ Elective percutaneous coronary intervention has revolutionized the outcome after acute myocardial infarction (MI) resulting in a remarkable decrease in in‐hospital heart failure.^2^ On the contrary, insufficient reestablishment of blood flow and late or unsuccessful reperfusion drive cardiomyocyte death, ventricular remodelling and increase in late heart failure prevalence. Consequently, despite advances in reperfusion, additional therapeutic strategies are an urgent medical need to improve the outcome after MI and to prevent progression to heart failure.

The progression of the chronic disease is characterized by complex mechanisms comprising changes in ventricular geometry and remodelling of the extracellular matrix.^3^ Cardiac fibroblasts (CFs) are critically involved in wound healing after MI and profibrotic processes. Transforming growth factor‐β (TGF‐β) is constitutively expressed in the heart in a latent form and becomes rapidly upregulated and released after MI. Subsequent TGF‐β activation by different mechanisms, involving, for example, matrix metalloproteinases (MMPs) or reactive oxygen species (ROS), triggers TGF‐β signalling cascades. Although being involved in the resolution of the inflammatory reaction in the infarcted MI, TGF‐β is also a strong inducer of fibrosis by stimulating the transdifferentiation of CFs into myofibroblasts thus increasing the expression of extracellular matrix (ECM) proteins, such as collagens.^4^, ^5^ CF activation and increased contractility of these cells improve wound healing and prevent infarct expansion and ventricular rupture after MI, but on the other, excessive deposition of ECM proteins may results in ventricular stiffness, reduced tissue compliance and consequently accelerated progression to heart failure.^6^ TGF‐β promotes an activated myofibroblast phenotype by binding to TGF‐β receptors, resulting in the activation of the canonical SMAD‐dependent pathway.^7^ SMAD‐independent, non‐canonical TGF‐β pathways, involving activation of mitogen‐activated protein kinases (MAPKs) and protein kinase B (Akt), have additionally been described.^8^ Increased levels of TGF‐β and activated TGF‐β signalling in CFs are hallmarks of cardiac fibrosis and are responsible for excessive ECM deposition.^9^

Peptidyl arginine deiminases (PADs) represent a family of Ca^2+^‐dependent enzymes consisting of five isozymes (PAD1‐4 and 6).^10^ Once activated, these enzymes catalyse the post‐translational modification of arginine to citrulline, thus regulating cell differentiation,^11^ apoptosis,^12^ neutrophil extracellular traps (NETs) formation,^13^ transcription^14^ and inflammation^15^ among others. Of note, PAD4 was thought to be the only PAD which could localize to the nucleus and, therefore, be involved in transcriptional regulation.^16^ In addition, PAD4 is critically involved in the release of NETs by activated neutrophils.^13^

Recently, CFs were found to express the isoforms PAD1, PAD2 and PAD4,^17^ and PAD4‐mediated regulation of TGF‐β signalling involving glycogen synthase kinase‐3β (GSK‐3β) has been reported in cancer cells.^18^ Moreover, using an established model of heart fibrosis, Martinod and colleagues found that PAD4 is associated with a profibrotic phenotype and contributes to organ fibrosis in mice.^19^ In this regard, the authors supposed that reduction of cardiac fibrosis in PAD4‐deficient mice exposed to ascending aortic constriction is due to compromised NETs formation and impaired NETs‐dependent transdifferentiation of fibroblasts into myofibroblasts.^19^, ^20^ Similarly, our group reported improved cardiac regeneration and ameliorated contractility in PAD4^‐/‐^ mice after myocardial infarction induced by permanent ligation of the left anterior descending artery.^21^ However, we hypothesize that abolished PAD4 activity is associated with impaired profibrotic responses resulting in attenuated adverse cardiac remodelling. Therefore, in the current study, we investigated the effect of PAD4 deficiency on the phenotypic and functional characteristics of primary isolated murine CFs and further assessed the activation of profibrotic pathways in response to TGF‐β.

## 2 MATERIALS AND METHODS

### 2.1 Echocardiography

WT and PAD4^‐/‐^ mice were housed at 22–24°C in a 12/24 h light/dark cycle under specific pathogen‐free conditions with food and water available ad libidum. PAD4‐deficient mice were generated as previously reported.^21^ All mice were on a C57BL/6J genetic background. Baseline cardiac function and dimensions in 9–12 weeks old WT (25.2 ± 2.28 g) and PAD4^‐/‐^ (25.5 ± 2.13) mice were assessed by transthoracic echocardiography as previously reported by our group.^21^ Mice were maintained under anaesthesia with continuously 2% isoflurane gas inhalation. Examination was conducted on a temperature‐controlled platform to ensure physiological heart and respiratory rates. Animal procedures were approved by the local animal care committee (Landesamt für Natur, Umwelt und Verbraucherschutz, Germany, No. 81‐02.04.2019.A318).

### 2.2 Isolation and culture conditions of CFs

Six to eight weeks old WT and PAD4^‐/‐^ mice were euthanized by cervical dislocation. CFs were isolated from excised hearts by enzymatic digestion and dissociation using GentleMACS Dissociator (Miltenyi Biotech). All procedures were approved by the local ethics committee (Bezirksregierung Köln; No: 4.19.007; 4.20.007). Briefly, hearts were placed in GentleMACS C tubes and digested by 100 U/ml collagenase II in Hanks‐buffered saline solution. Cell suspensions were filtered through 70 µm cell strainers and plated in T75 flasks in Dulbecco's Modified Eagle Medium: Nutrient Mixture F12 (DMEM/F12) medium supplemented with 10% FCS and 100 U/ml penicillin and 10 µg/ml streptomycin (Sigma‐Aldrich) at a density of 5–10 × 10^6^ cells. For subculture, cells were seeded into gelatin‐coated flasks or plates. Fibroblasts at passage 3 (P3) were used in all experiments. Cells were cultured in serum‐reduced medium (1% or 0.2% FCS) for 24 h before experiments. When indicated, the PAD4 inhibitor Cl‐amidine (Cayman Chemical) was added to the culture medium. Then, CFs were treated with TGF‐β for different times.

### 2.3 Proliferation assay

Cell proliferation was assessed using a chemiluminescence‐based BrdU Cell Proliferation Elisa Kit (Roche Applied Biosciences). 2500 CFs from WT and PAD4^‐/‐^ mice were seeded in 96‐well plates for 24 h and further cultured in low‐serum DMEM/F12 (1% FCS) for additional 24 h. After stimulation with 10 ng/ml TGF‐β (R&D Systems) for 24 h, 10 µM BrdU was added to the wells. BrdU incorporation was detected after 20 h by immunohistochemical staining using anti‐BrdU antibodies and peroxidase‐conjugated secondary antibodies. All samples were run in triplicates.

### 2.4 Collagen gel contraction

CFs were cultured in low‐serum medium (DMEM/F12 + 0.2% FCS) for 24 h prior experiments. Cells were detached using 0.05% Trypsin/EDTA solution and finally resuspended at a density of 6 × 10^5^/ml. Collagen matrix was prepared on ice by diluting a stock solution of 3 mg/ml rat collagen I (Thermo Fisher) with 2 × DMEM for a final concentration of 1.3 mg/ml collagen and combined with CFs of either PAD4^‐/‐^ or WT mice to yield a final concentration of 2 × 10^5^ cells/ml. Subsequently, 500 μl of this suspension was plated into 24‐well culture plates and allowed to polymerize at 37ºC for 60 min. Following polymerization, pads were released from wells, transferred to 6‐well culture plates and cultured in DMEM/F12 + 0.2% FCS and 10 ng/ml TGF‐β1 (R&D Systems). At 0 h, 24 h and 48 h, the area of each gel was analysed with an image analyzer (ChemiDoc XRS+System, Bio‐Rad). Data are expressed as the percentage of area compared with the initial gel area. Two replicates were analysed per condition.

### 2.5 Wound healing assay

To determine the relative migratory ability of CFs, cells (2 × 10^4^) were cultured in gelatin‐coated 6‐well plates until 80%–90% confluence. To avoid cell proliferation, medium was replaced by low‐serum culture medium containing 0.2% FCS and cells were further cultured for 24 h before the monolayer was scratched with a blue pipet tip. After cells were gently washed with PBS, TGF‐β (10 ng/ml) was added to each well and culture was continued for 24 h. The scratch wounds were photographed at the time the scratch was created and 24 h later. The relative migration of CFs into the denuded area was measured using ImageJ software (National Institutes of Health). Migration experiments were performed in triplicate.

### 2.6 Real‐time PCR

RNA was extracted from excised hearts of WT and PAD4^‐/‐^ mice or cultured CFs using TRIzol (Invitrogen) and the RNeasy Mini Kit (Qiagen), respectively. Reverse transcription to cDNA was performed using High Capacity cDNA Reverse Transcription Kit (Applied Biosystems). Real‐time PCR was performed using PowerUp SYBR green PCR master mix (Applied Biosystems) QuantStudio 3 Real‐Time PCR System (Applied Biosystems). The following primer pairs specific for mouse were used: Collagen I forward: 5′‐GTACTCCTGGTGCTGATG‐3′, reverse: 5′‐GAAGCCTCTTTCTCCTCTCTGA‐3′; Collagen III forward: 5′‐GCCCACAGCCTTCTACAC‐3′, reverse: 5′‐CCAGGGTCACCATTTCTC‐3′; α‐SMA forward: 5′‐GGCTCTGGGCTCTGTAAGG‐3′, reverse: 5′‐CTCTTGCTCTGGGCTTCATC‐3′; MMP‐2 forward: 5′‐TTCCCCCGCAAGCCCAAGTG‐3′, reverse: 5′‐GAGAAAAGCGCAGCGGAGTGACG3′; MMP‐9 forward: 5′‐TCACCTTCACCCGCGTGTA‐3′, reverse 5′‐GTCCTCCGCGACACCAA‐3′; TGF‐β forward: 5′‐TGACGTCACTGGAGTTGTACGG‐3′, reverse: 5′‐GGTTCATGTCATGGATGGTGC‐3′; GAPDH forward: 5′‐CGACTTCAACAGCAACTCCCACTCTTCC‐3′, reverse 5′‐TGGGTGGTCCAGGG‐TTTCTTACTCCTT‐3′. All samples were run in triplicate and normalized to GAPDH gene expression. Data are expressed as 2^‐ΔCT^.

### 2.7 Subcellular fractionation

Preparation of nuclear extracts was performed using the NE‐PER Nuclear and Cytoplasmic Extraction Kit (Thermo Fisher) according to manufacturer's instructions. In brief, 1 × 10^6^ CFs were suspended in 100 µl CER‐I buffer followed by the addition of 5.5 µl buffer CER‐II and vigorous vortex of samples. The supernatant was harvested as the cytoplasmic fraction. The pellet was washed twice and further resuspended in 40 µl Nuclear extraction buffer NER and vortexed on the highest setting. After centrifugation, the supernatants were collected as the nuclear fraction and stored at ‐80°C.

### 2.8 Western blotting

Total protein was extracted from hearts or cultured cells using RIPA buffer supplemented with protease and phosphatase inhibitors (Cell Signaling Technology). Equal amounts of protein were loaded onto SDS‐polyacrylamide gels and blotted onto nitrocellulose membranes (Bio‐Rad). After blocking, membranes were incubated with the following primary antibodies overnight at 4°C: Collagen I (PA5‐29569, Thermo Scientific, 1:1000), Collagen III (PA5‐27828, Thermo Scientific, 1:1000), α‐SMA (14968, Cell Signaling, 1:1000), pSMAD2 (3108, Cell Signaling, 1:1000), SMAD2 (5339, Cell Signaling, 1:1000), pSMAD3 (NBP1‐77836SS, Novus, 1:2000), SMAD3 (9513, Cell Signaling, 1:1000), pSTAT3 (9145, Cell Signaling, 1:1000), STAT3 (4904T, Cell Signaling, 1:2000), pGSK‐3β (9323T, Cell Signaling, 1:1000), GSK‐3β (12456, Cell Signaling, 1:1000), pAkt (9271, Cell Signaling, 1:1000), Akt (4691, Cell Signaling, 1:1000), pERK1/2 (9101, Cell Signaling, 1:1000) and ERK1/2 (9102, Cell Signaling, 1:1000).

After incubation with HRP‐conjugated secondary antibodies (Dako), membranes were developed with UptiLight HRP Blot Chemiluminescent ECL Substrate (Uptima). The density of bands was quantified using Image Lab software (Bio‐Rad).

For normalization and to confirm equal loading, blots were re‐probed with antibodies against GAPDH (NBP2‐27103, Novus, 1:5000), β‐actin (4967, Cell Signaling, 1:1000) or Histon H3 (4499, Cell Signaling, 1:2000). For normalization of pAkt, pERK1/2, pSMAD2, pSMAD3, pSTAT3 and pGSK‐3β, total protein was used. Uncropped Western blot images are shown in Figure S1.

### 2.9 Histology and immunofluorescence

Murine hearts were fixed in 4% formaldehyde, embedded in paraffin and cut into 4‐µm sections. Masson trichrome staining of heart tissue sections was used to detect cardiac fibrosis. For immunofluorescence, sections were deparaffinized and rehydrated, and antigen retrieval was performed using citrate buffer, pH 6 (Sigma‐Aldrich). In brief, sections were permeabilized and blocked in 5% goat serum and 0.3% TritonX‐100 in PBS and incubated with polyclonal antibodies against α‐SMA (1:1000, Abcam) overnight at 4°C. Then, sections were washed in PBS and incubated with Alexa Fluor 488‐conjugated goat anti‐rabbit IgG (Cell Signaling) followed by incubation with DAPI. Fluorescence images were acquired using a Nikon Eclipse Ti‐U 100 inverted microscope.

### 2.10 Statistical analyses

Investigators performing echocardiographic analyses were blinded with respect to mice genotype. Statistical analyses were performed using GraphPad Prism software (GraphPad Software Inc.). Data are presented as mean ± standard deviation.^22^ Data sets were assessed for normality using the Kolmogorov–Smirnov test. Normally distributed unpaired data of multiple groups were analysed using one‐way ANOVA followed by Newman Keuls or Dunnett's post hoc test. The two‐tailed unpaired *t*‐test was used for 2‐group comparison. *p*‐values < 0.05 were considered as statistically significant.

## 3 RESULTS

### 3.1 Impact of peptidylarginine deiminase 4 deficiency on cardiac function

**FIGURE 1:**
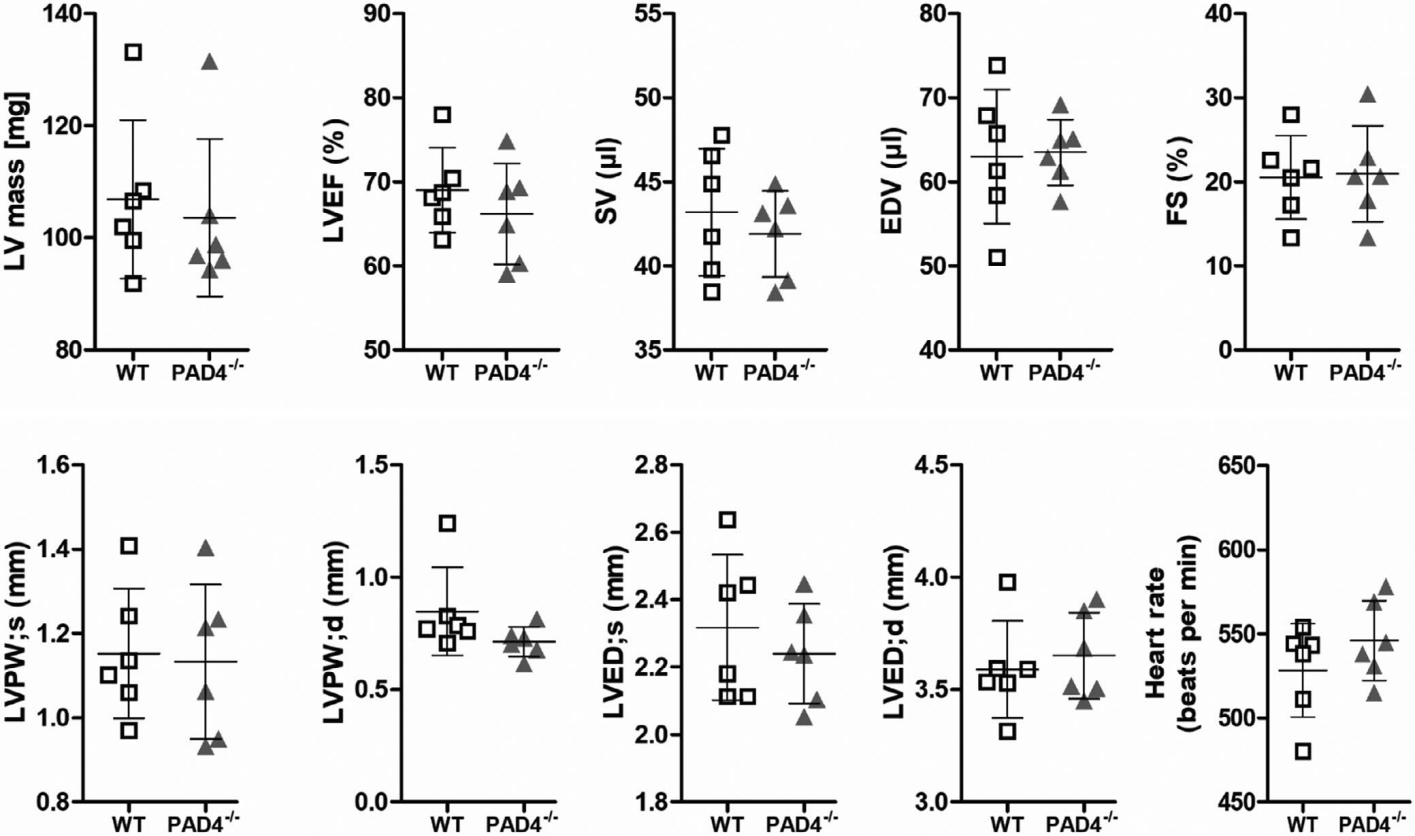
Comparison of cardiac function and dimensions between WT and PAD4^−/−^ mice at baseline. Left ventricular (LV) mass and echocardiographic analysis of ejection fraction (EF), stroke volume (SV), end‐diastolic volume (EDV), fractional shortening (FS), left ventricular posterior wall thickness (LVPW), left ventricular end‐diastolic diameter (LVED) and heart rate under baseline conditions. *n* = 6/group

To prove whether PAD4 deficiency influences cardiac function in mice, we first performed transthoracic echocardiographic analyses on WT and PAD4^‐/‐^ mice. At baseline, echocardiographic parameters like heart rate, ejection fraction (EF), stroke volume (SV), fractional shortening (FS) and end‐diastolic volume (EDV) in PAD4^‐/‐^ mice were comparable with those in WT mice (Figure 1). Additionally, no differences in heart mass and dimensions were detected.

### 3.2 Expression of fibrosis markers in peptidylarginine deiminase 4‐deficient murine hearts

**FIGURE 2:**
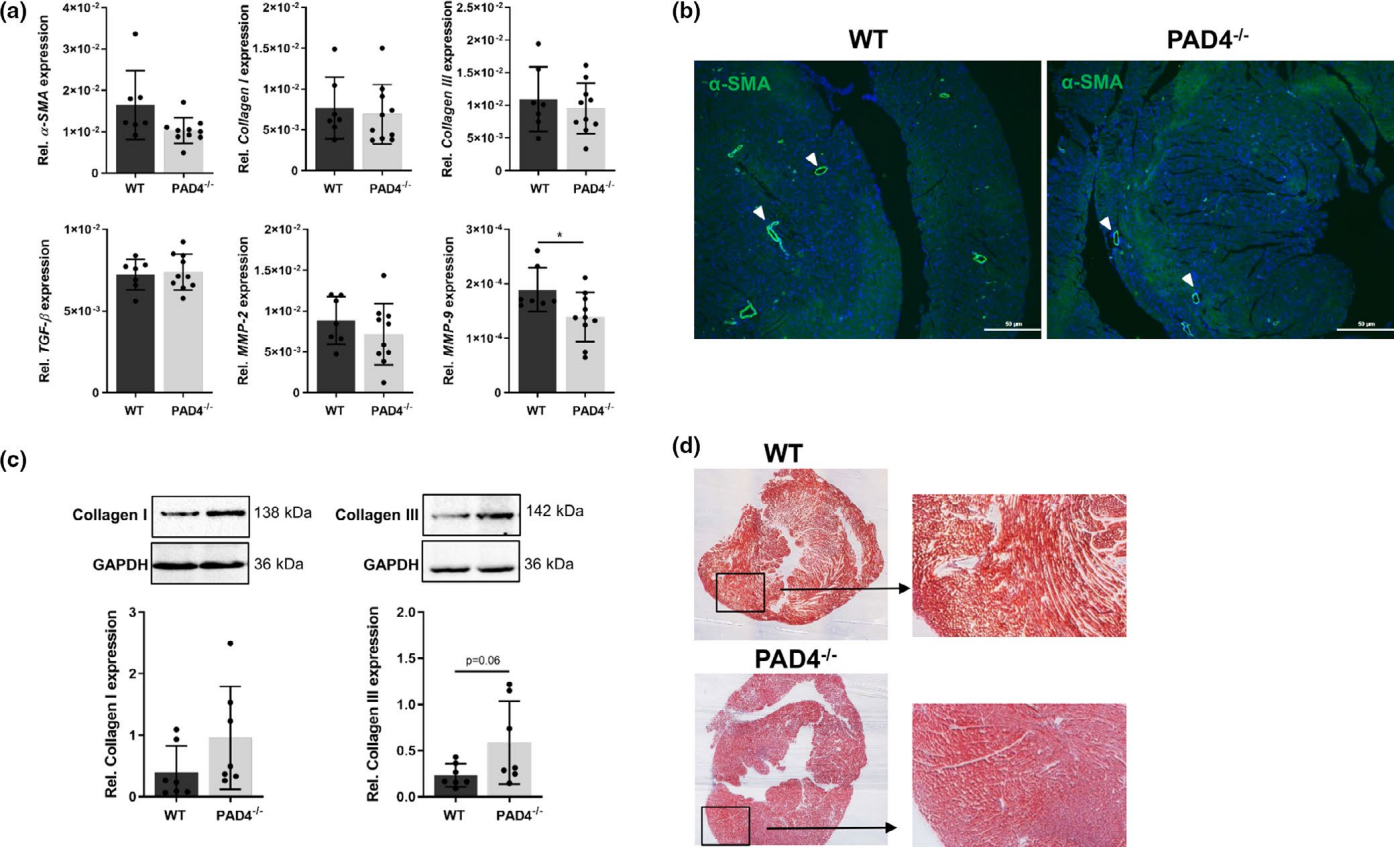
Expression of fibrosis‐related markers in PAD4^−/−^ hearts. (A) The expression of fibrosis‐related genes in WT (*n* = 7) and PAD4^−/−^ (*n* = 10) hearts was analysed by real‐time PCR. (B) α‐SMA expression was detected by immunofluorescence and was found to be restricted to vascular cells (*white arrow heads*). One representative image of three stained hearts is depicted. Scale bar indicates 100 µm. *n* = 5/group. (C) Collagen I and III protein expression in cardiac tissue of WT and PAD4^−/−^ mice. *n* = 7. (D) Representative images of heart sections from WT and PAD4‐deficient mice stained with Masson trichrome under baseline conditions. *p < 0.05

PAD4 deficiency has previously been described to protect against organ fibrosis.^19^, ^23^ Therefore, we next investigated whether there are any differences in the expression of fibrosis markers between WT and PAD4^‐/‐^ hearts. Whereas the expression of *α*‐*smooth muscle actin (α*‐*SMA)*, *collagen I*, *collagen III*, *TGF*‐*β* and *MMP*‐*2* did not differ between the different genotypes, significant downregulation of *MMP*‐*9* could be detected in PAD4^‐/‐^ hearts (Figure 2A). Immunofluorescence staining showed cardiac α‐SMA expression solely restricted to vascular endothelial cells (Figure 2B), and no α‐SMA‐positive CFs were detected, suggesting that these cells do not transdifferentiate into myofibroblasts under baseline conditions. However, cardiac collagen type I and type III protein expression were clearly, but not significantly elevated in PAD4^‐/‐^ hearts (Figure 2C,D). In addition, the expression of SMAD3 and STAT3, which are critically involved in fibrotic processes,^24^, ^25^ was similar between both genotypes (Figure S2).

### 3.3 Characterization of cardiac fibroblasts in culture

**FIGURE 3:**
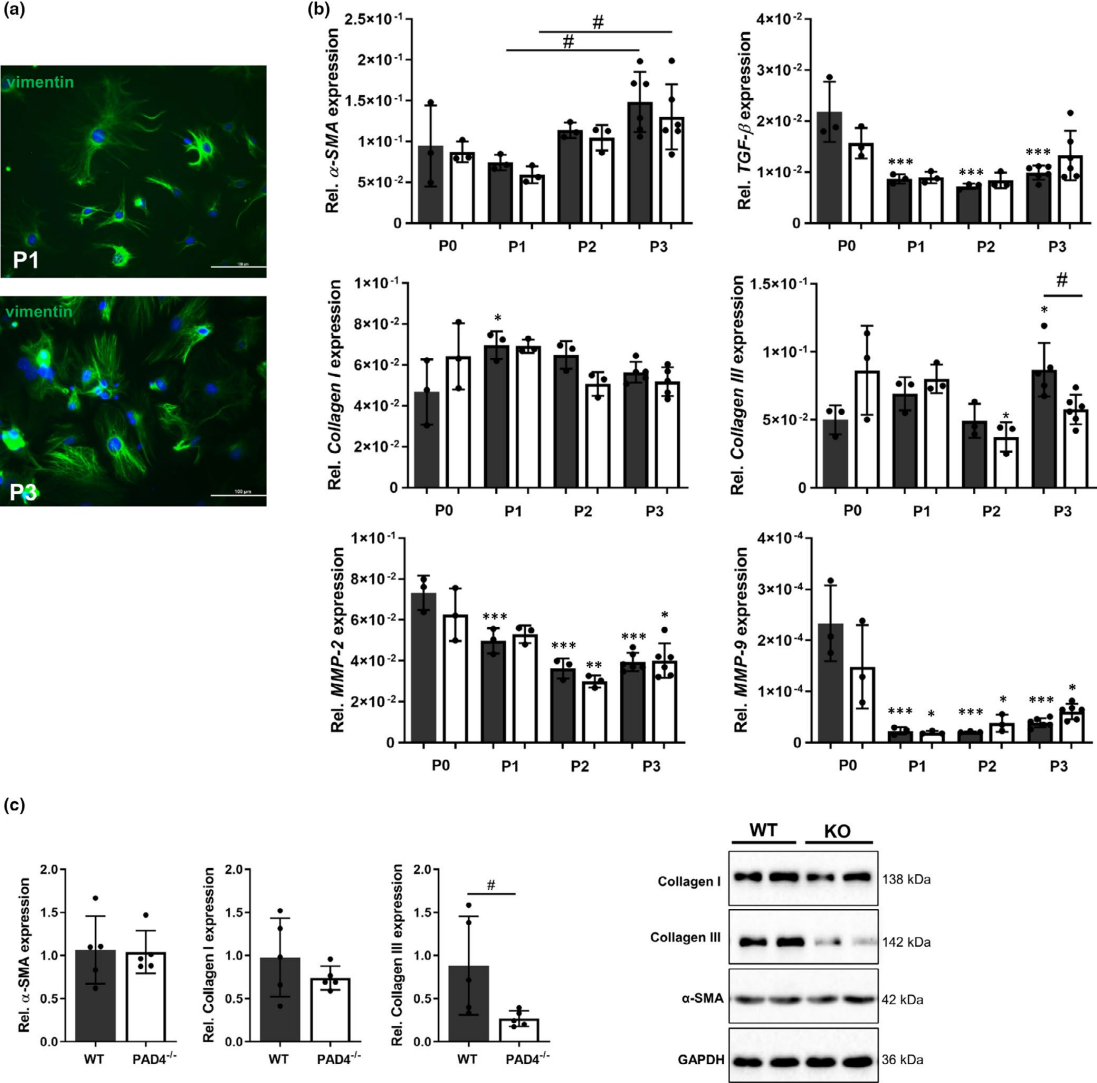
Characterization of cardiac fibroblasts (CFs) in culture. CFs were isolated from WT and PAD4^−/−^ hearts and cultured until passage (P) 3. (A) At P1 and P3, all CFs were positive for vimentin. One representative image is depicted. Scale bar indicates 100 µm. (B) Gene expression of *α‐SMA* gradually increased in cultured WT and PAD4^−/−^ CFs. *TGF‐β*, *MMP‐2* and *MMP‐9* expression strongly decreased after the first passage. Whereas *collagen I* expression was constant, *collagen III* gene expression declined in PAD4^−/−^ CF and was significantly reduced at P3 when compared to WT CFs. *n* = 3–6/group. (C) At P3, reduced collagen III protein expression in PAD4^−/−^ CFs was detected and no alteration in α‐SMA and collagen I levels. *n* = 5/group *p < 0.05, **p < 0.01,***p < 0.001 vs P0 for B. ^#^ *p* < 0.05 for B and C

To verify molecular phenotypic differences between WT and PAD4^‐/‐^ CFs as well as their ability to spontaneously transdifferentiate into myofibroblasts, freshly isolated cells were cultured for three passages in parallel. Both, cardiac fibroblasts at P1 and P3 were found to be positive for the fibroblast marker vimentin (Figure 3A). The expression of *TGF*‐*β*, *MMP*‐*2* and *MMP*‐*9* strongly decreased during culture but was not affected by PAD4 deficiency (Figure 3B). As already described in the literature,^26^ *α*‐*SMA* expression gradually increased from P1 to P3, suggesting that the cells spontaneously differentiate into myofibroblasts under *ex vivo* culture conditions. At P0, gene expression of collagens tended to be higher in PAD4^‐/‐^ CFs and further significantly changed during subculture. WT cells significantly upregulated *collagen III* expression during *in vitro* culture. In contrast, P3 PAD4^‐/‐^ CFs displayed significant reduction of collagen III gene and protein expression and comparable levels of α‐SMA and collagen I (Figure 3B,C). In general, no morphological changes could be observed in PAD4^‐/‐^ CFs.

### 3.4 Peptidylarginine deiminase 4 deficiency alters functional characteristics of cardiac fibroblasts in response to transforming growth factor‐β

**FIGURE 4:**
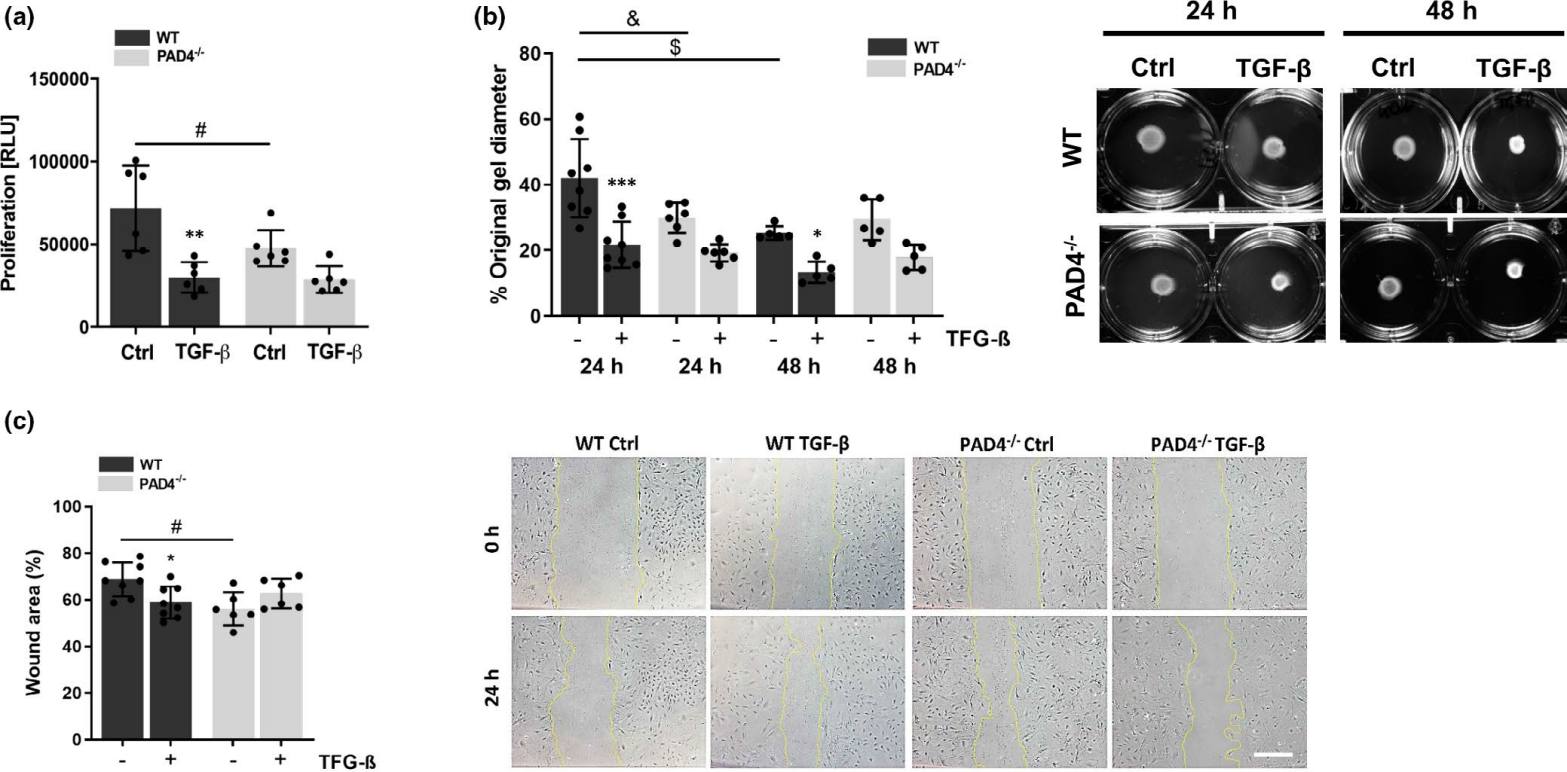
Functional differences between WT and PAD4^−/−^ CFs in response to TGF‐β treatment. (A) CFs were stimulated with TGF‐β (10 ng/ml) for 24 h. Cell proliferation was assessed by BrdU incorporation after 20 h of labelling. Exposure to TGF‐β significantly reduced the proliferation of WT CFs. PAD4^−/−^ CFs displayed reduced proliferation rate in control samples, and no effect of TGF‐β was detected. *n* = 6. (B) CFs were cultured in a collagen gel and stimulated with TGF‐β (10 ng/ml) for 48 h. TGF‐β significantly enhanced collagen gel contraction by WT CFs after 24 h and 48 h. Contrary, TGF‐β‐stimulated contraction of PAD4^−/−^ CFs was markedly impaired. Collagen gel contraction in unstimulated cells (without TGF‐β) was increased in PAD4^−/−^ CFs after 24 h and in WT cells after 48 h when compared to control WT cells (24 h), corresponding to reduced gel diameters (*left panel*). One representative experiment is displayed (*right panel*). *n* = 6–8. (C) Migration of WT CFs in wound healing assay was increased by TGF‐β treatment for 24 h. Unstimulated PAD4^−/−^ CFs displayed accelerated wound closure, and no effect of TGF‐β could be observed (*left panel*). Representative images of one experiment are depicted (*right panel*). Scale bar indicates 100 µm. *n* = 5–8. *p < 0.05, **p < 0.01, ***p < 0.001 vs. control cells; ^#^ *p* < 0.05, ^&^ *p* < 0.01, ^$^ *p* < 0.001

To study the effects of PAD4 deficiency on fibroblast function, we first assessed the proliferation, contraction and migration of PAD4^‐/‐^ CFs. TGF‐β is known to provide myofibroblast differentiation and fibroblast contraction, and both, pro‐ and anti‐proliferative effects on CFs, were already described *in vitro*.^24^, ^27^, ^28^ Cells were medium‐starved before experiments. Initial experiments performed by our group using different concentrations of TGF‐β (2 ng/ml; 5 ng/ml; 10 ng/ml) showed dose‐dependent effects of TGF‐β. Because the most prominent results were achieved using the highest concentration of TGF‐β, 10 ng/ml was chosen for further experiments. As shown in Figure 4A, TGF‐β significantly reduced the proliferation of WT CFs but not of PAD4^‐/‐^ cells. Proliferation of PAD4^‐/‐^ CFs was significantly diminished after 24 h in culture. By performing collagen gel contraction assay, we found that TGF‐β induced significant gel contraction in WT CFs after 24 and 48 h, respectively (Figure 4B). Of note, contraction of PAD4‐deficient CFs was markedly attenuated under same experimental conditions. After 24 h in culture, these cells showed significantly increased contraction when compared to WT cells, suggesting increased contractility of PAD4^−/−^ CFs in the absence of TGF‐β. Extensive contraction of WT CFs was seen after 48 h in culture when compared to gel size determined after 24 h. Further, cell migration during the wound healing process was significantly accelerated in TGF‐β‐treated WT CFs but no effect could be observed in PAD4‐deficient CFs (Figure 4C). Again, spontaneous migratory activity of PAD4‐deficient CFs was significantly higher when compared to WT cells. Taken together, these results demonstrate that PAD4^‐/‐^ CFs cultured *ex vivo* display spontaneous reduction in cell proliferation, increased contractility and migratory activity in wound healing assay, combined with impeded responsiveness to TGF‐β.

It has previously been reported that PAD4 inhibition in breast cancer cells results in diminished nuclear localization of GSK‐3β and consequently activated TGF‐β signalling.^18^ Overexpression of GSK‐3β was further reported to promote SMAD3 degradation desensitizing cells to TGF‐β.^29^ We therefore proved the subcellular localization of GSK‐3β in unstimulated, cultured WT and PAD4^‐/‐^ CFs. However, in our experimental setting, we could not detect any differences in nuclear GSK‐3β and SMAD3 protein levels (Figure S3), suggesting that nuclear levels of GSK‐3β do not seem to be responsible for increased activity of PAD4^‐/‐^ CFs under baseline conditions.

### 3.5 Peptidylarginine deiminase 4 deficiency impairs activation of the non‐canonical transforming growth factor‐β pathway and transdifferentiation into myofibroblasts

**FIGURE 5:**
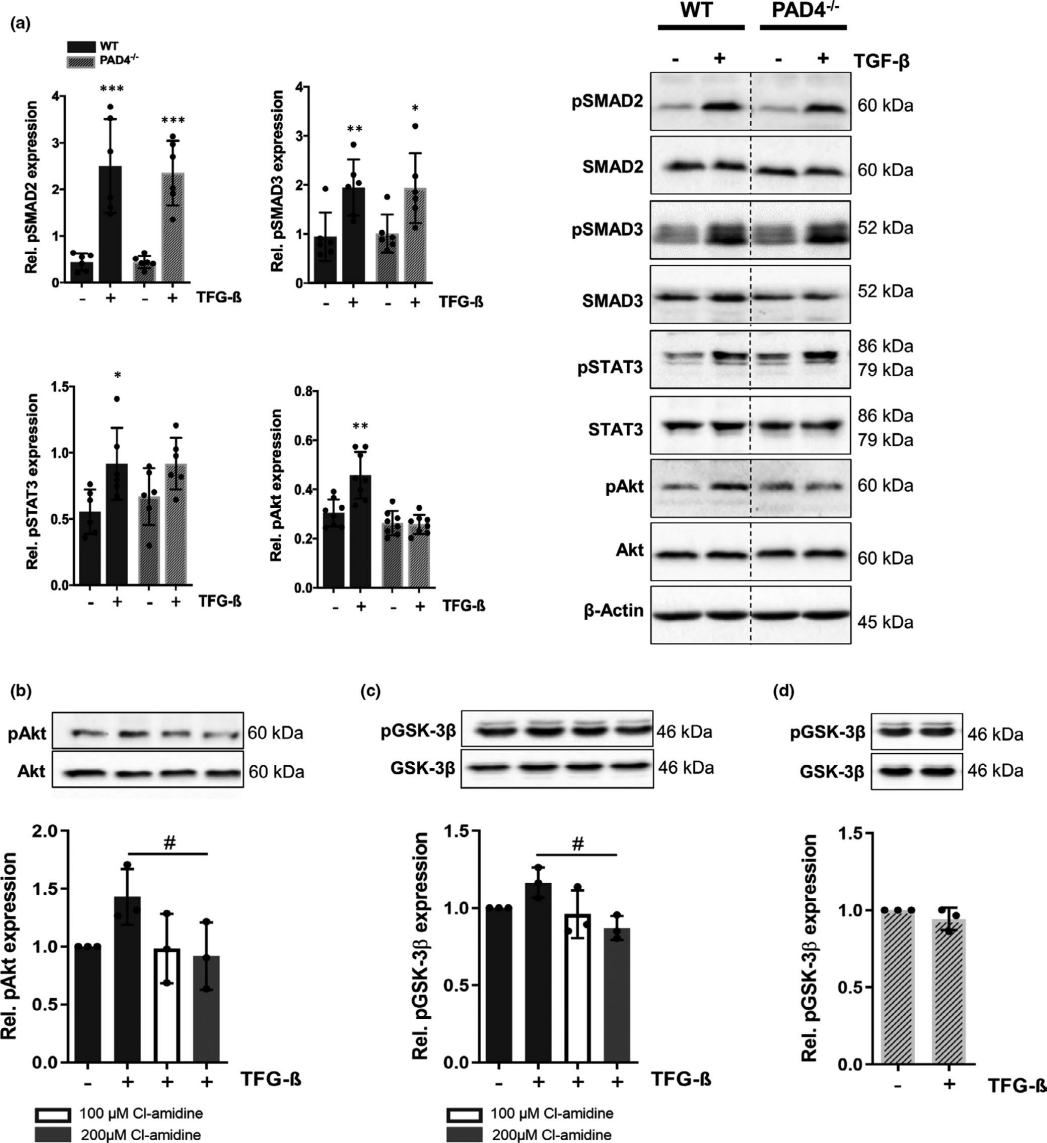
Impact of PAD4 deficiency on TGF‐β signalling. (A) Activation of the canonical and non‐canonical pathway by TGF‐β was examined by Western blot. TGF‐β stimulation (10 ng/ml) for 2 h significantly increased protein levels of pSMAD2 and pSMAD3 in CFs independently on PAD4 deficiency. TGF‐β failed to activate STAT3 and Akt in PAD4^−/−^ CFs. *n* = 6–8. Preincubation of WT CFs with the PAD4 inhibitor Cl‐amidine for 18 h abrogated TGF‐β‐induced activation of Akt (B) and subsequent GSK‐3β phosphorylation (C). *n* = 3. (D) No TGF‐β‐triggered phosphorylation of GSK‐3β was detected in PAD4^−/−^ CFs. *n* = 3. *p < 0.05, **p < 0.01, ***p < 0.001 vs. control cells for A; ^#^ *p* < 0.05 for B and C

To examine whether PAD4 deficiency is associated with an impaired activation of TGF‐β‐induced signalling pathways, we studied the phosphorylation status of SMAD proteins and SMAD‐independent pathways including signal transducer and activator of transcription 3 (STAT3),^30^ extracellular signal‐regulated protein kinases 1 and 2 (ERK1/2) and Akt^8^. Stimulation of cells with TGF‐β induced pronounced upregulation of phosphorylated SMAD2 (pSMAD2) and pSMAD3 in both, WT and PAD4^‐/‐^ CFs. Importantly, no TGF‐β‐mediated activation of Akt could be seen in PAD4^‐/‐^ CFs and only a minor, not significant, increase in pSTAT3 levels was found (Figure 5A). No ERK1/2 activation could be detected in our experiments (not shown). Of note, inhibition of PAD4 activity in WT CFs by Cl‐amidine prevented Akt phosphorylation, confirming that PAD4 activity is required for TGF‐β‐mediated activation of the non‐canonical pathway (Figure 5B). As recent findings have suggested that Akt activation is accompanied by phosphorylation of GSK‐3β on Ser9,^31^ we tested whether TGF‐β‐stimulated CFs display reduced levels of phosphorylated GSK‐3β. Akt‐mediated GSK3‐β phosphorylation is associated with kinase inactivation and upregulation of TGF‐β signalling. Indeed, chemical PAD4 inhibition abrogated upregulation of pGSK‐3β (Figure 5C). Similarly, PAD4^‐/‐^ CFs exposed to TGF‐β did not upregulate pGSK‐3β levels (Figure 5D). Thus, suppressed Akt activation seems to be accompanied by increased GSK‐3β activity in these cells.

**FIGURE 6:**
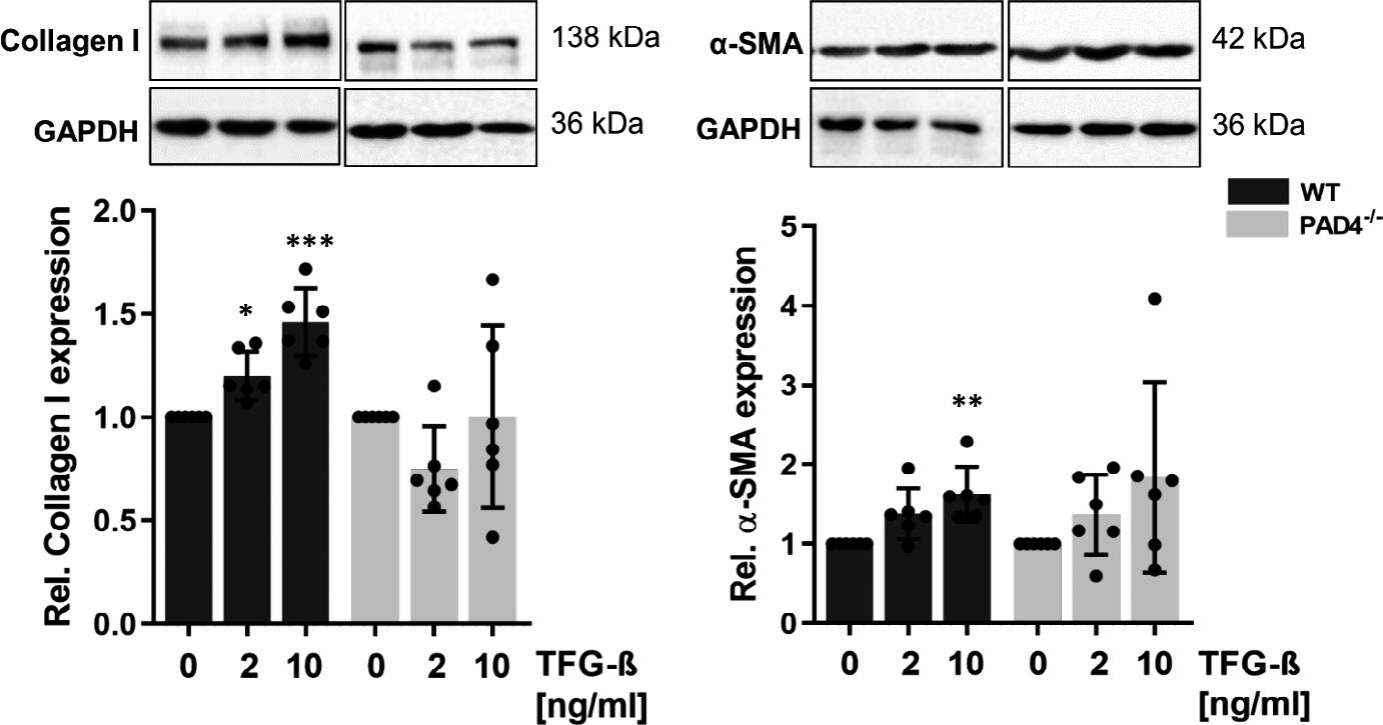
Effect of PAD4 deficiency on TGF‐β‐induced upregulation of fibrotic markers. WT and PAD4^−/−^ CFs were exposed to TGF‐β (2 ng/ml and 10 ng/ml) for 48 h. The expression of collagen I and α‐SMA was assessed by Western blot. PAD4^−/−^ CFs failed to upregulate collagen I and α‐SMA protein expression. *n* = 6/group. *p < 0.05, **p < 0.01, ***p < 0.001 vs. unstimulated control cells

Additional experiments revealed that impaired TGF‐β signalling in PAD4^‐/‐^ CFs markedly reduces the ability of cells to produce matrix proteins. As shown in Figure 6, WT CFs upregulated collagen type I expression after TGF‐β stimulation in a concentration‐dependent manner. At the same time, α‐SMA upregulation, indicating transdifferentiation into myofibroblasts, could be demonstrated. In contrast, PAD4‐deficient CFs were unable to significantly upregulate α‐SMA and collagen I protein expression in response to TGF‐β, confirming deregulated TGF‐β signalling in these cells.

## 4 DISCUSSION

In this study, we found that PAD4 is critically involved in the regulation of TGF‐β signalling in primary CFs. We demonstrate that in the absence of PAD4, both, CF function and collagen production in response to TGF‐β are significantly reduced due to impaired activation of the non‐canonical pathways involving Akt and STAT3.

The enzyme PAD4 targets multiple proteins involved in gene regulation,^14^ apoptosis,^12^ NETosis,^32^ inflammation^15^ and pluripotency.^33^ Data presented herein demonstrate that PAD4 is a notable regulator of TGF‐β signalling pathways in CFs and loss of function significantly diminishes fibrotic responses. Under baseline conditions, we found collagen I and III expression to be visibly elevated in PAD4^‐/‐^ murine hearts as well as on mRNA level in isolated CFs at an early passage (P0). Of note, *MMP*‐*2* and ‐*9* expression tended to be lower in P0 PAD4^‐/‐^ CFs, and *MMP*‐*9* expression was significantly reduced in PAD4‐deficient hearts, raising the possibility of an imbalanced collagen homeostasis in PAD4^‐/‐^ mice. However, these changes in collagen and MMP expression in PAD4^‐/‐^ hearts did not affect baseline cardiac function or dimensions, respectively. This finding is in line with current reports showing that compromised cardiac expression of matrix proteins or MMP‐9 does not mandatory induce a cardiac phenotype without stress.^34^, ^35^ Absence of cardiac phenotype in mice lacking PAD4 activity might additionally be explained by functional SMAD signalling. In this regard, activation of SMADs, including SMAD2 and 3, by phosphorylation was found to be required for cardiogenesis.^36^, ^37^ Besides, numerous studies illustrated that MMPs are essential to migration in and remodelling of the ECM.^38^, ^39^ As PAD4‐dependent citrullination of collagen I was described to improve cell adhesion and to decrease cell motility,^40^ altered post‐translational modification of ECM in PAD4^‐/‐^ mice may influence cardiac recruitment of cells at least under pathological conditions.

During *in vitro* culturing, CFs strongly reduced *TGF*‐*β* and *MMP*s expression and significantly upregulated *α*‐*SMA* mRNA levels, independently of the genotype. These findings are in agreement with prior studies reporting spontaneous differentiation of CFs into an activated phenotype *in vitro* ^26^ and additionally demonstrate that the transcriptome of CFs changes already after the first passage making it challenging to study their functional characteristics *in vitro*. Here, WT and PAD4^‐/‐^ CFs were cultured in parallel under identical culture conditions. At passage 3, we did not observe any differences in fibrotic markers between WT and PAD4^‐/‐^ CFs except for collagen III, which was substantially diminished in PAD4^‐/‐^‐deficient cells.

Our functional studies revealed that PAD4^‐/‐^ CFs showed increased migratory capacity and collagen contraction as well as reduced proliferation after 24 h in culture. Similar results were obtained using WT CFs exposed to TGF‐β, whereas TGF‐β treatment of PAD4^‐/‐^ CFs did not affect their function and proliferation. We therefore speculated that PAD4 deficiency might be associated with a baseline activation of TGF‐β signalling pathways, which could possibly also explain impaired TGF‐β responsiveness in these cells. In this context, PAD4‐dependent citrullination and nuclear translocation of GSK‐3β resulting in the activation of TGF‐β signalling in human breast cancer cells have previously been demonstrated.^18^ Although no further studies exist demonstrating a link between PAD4 and TGF‐β signalling, it becomes apparent that additional regulatory mechanisms might be involved, as similar nuclear levels of GSK‐3β and SMAD3 were detected in WT and PAD4^‐/‐^ CFs under baseline conditions. However, on the basis of our results suggesting similar α‐SMA expression in PAD4‐deficient CFs, a baseline activation of fibrotic pathways in PAD4^‐/‐^ CFs cannot be entirely excluded.

Further investigation revealed that PAD4 deficiency abrogates TGF‐β‐triggered phosphorylation of Akt and GSK‐3β, which play important roles in the pathology of fibrosis.^25^, ^31^ STAT3 activation was also markedly impaired in PAD4^‐/‐^ CFs. GSK‐3β was reported to directly interact with SMAD3, thus negatively regulating protein stability. In turn, overexpression of GSK‐3β promoted SMAD3 degradation and desensitized cells to TGF‐β.^29^ Although the conical, SMAD‐dependent TGF‐β signalling pathway was undisturbed in PAD4^‐/‐^ CFs, there was a prominent decrease in TGF‐β‐triggered fibrotic responses including α‐SMA and collagen I upregulation, indicating a significant contribution of Akt and probably STAT3 signalling to fibrosis. Similarly, Qu et al.^28^ reported that myofibroblast transdifferentiation critically depends on functional Akt/GSK3β signalling. In addition, STAT3 was identified as a crucial mediator of fibrosis, and inactivation of STAT3 was reported to inhibit TGF‐β‐induced myofibroblast differentiation and collagen release in cultured dermal fibroblasts.^25^ However, the underlying mechanisms by which PAD4 regulates the activity of Akt and STAT3 were not specifically studied here and still remain elusive. Whether STAT3 becomes directly regulated by PAD4 in CFs or rather represents a downstream target of pI3K/Akt^41^, ^42^ remains an open question.

Several reports in the past found that PAD4 inhibition attenuates organ fibrosis^19^ and protects against cardiac dysfunction after MI.^43^, ^44^ The protective effects were attributed to the deficiency of NETs whereby additional cellular and molecular aspects were largely neglected. In the light of our findings, we suggest that protection against cardiac dysfunction after MI based on PAD4 inhibitors or PAD4 deficiency substantially depends on abrogated TGF‐β signalling in CFs. After MI, increased TGF‐β expression promotes CF activation and increased production of ECM proteins. This initial step is critical to prevent infarct expansion and cardiac rupture. As inhibition of TGF‐β is known to prevent late remodelling after MI,^45^ compromised TGF‐β signalling in PAD4^‐/‐^ CFs might partially explain improved cardiac compliance and function after MI^21^ as well as reduced organ fibrosis.^19^, ^21^, ^23^ Previous as well as unpublished data of our group revealed reduced cardiac remodelling in PAD4^‐/‐^ mice subjected to MI, as evidenced by decreased end‐diastolic volume and diameter, increased contractility and EF.^21^ However, because we did not investigate the impact of PAD4 deficiency on other cardiac cell types, such as cardiomyocytes, additional aspects may merit consideration. In this context, the implication of PAD4 in cardiomyocyte survival and protection should be critically evaluated as the pathways are not fully explored.

In summary, this study is, to our knowledge, the first demonstrating that PAD4 is involved in the regulation of TGF‐β‐induced profibrotic pathways. Consequently, PAD4 should be considered as a target for pharmaceutical intervention to prevent cardiac fibrosis. Nevertheless, further *in vivo* studies are required to assess translational relevance of the current findings.

## CONFLICT OF INTEREST

The authors confirm that there are no conflicts of interest.

## AUTHOR CONTRIBUTIONS

**Hanane Akboua:** Formal analysis (lead); Investigation (lead); Validation (equal). **Kaveh Eghbalzadeh:** Funding acquisition (lead); Validation (supporting); Writing‐review & editing (supporting). **Ugur Keser:** Investigation (supporting); Methodology (supporting). **Thorsten Wahlers:** Supervision (equal); Writing‐review & editing (equal). **Adnana Paunel‐Görgülü:** Conceptualization (lead); Supervision (lead); Validation (equal); Writing‐original draft (lead).

## Supporting information

Fig S1

Fig S2

Fig S3

## Data Availability

All data generated or analysed in this study are available on request from the corresponding author.
