# Supplementary material for: Impaired non‐canonical transforming growth factor‐β signalling prevents profibrotic phenotypes in cultured peptidylarginine deiminase 4‐deficient murine cardiac fibroblasts
Source: J Cell Mol Med. 2021 Sep 14;25(20):9674–84. doi: 10.1111/jcmm.16915 (PMC8505821; doi:10.1111/jcmm.16915)
Supplement: Supplementary file 1 — Fig S1 [file JCMM-25-9674-s002.pdf]

Impaired non-canonical TGF-β signaling prevents profibrotic phenotypes in cultured PAD4-deficient murine cardiac fibroblasts

Hanane Akboua[1], Kaveh Eghbalzadeh[1], Ugur Keser[1], Thorsten Wahlers[1], Adnana Paunel-Görgülü[1*]

[1]Department of Cardiothoracic Surgery, Heart Center of the University of Cologne, Cologne, Germany

Supplementary Figure 1:

Figure 2:

Collagen I

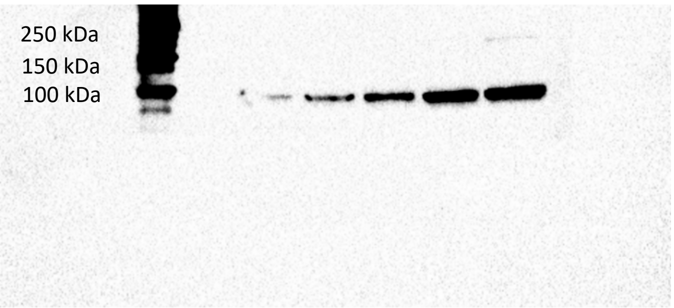

GAPDH

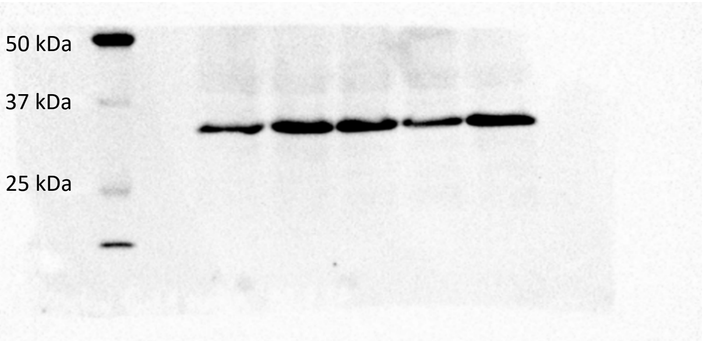

Collagen 3

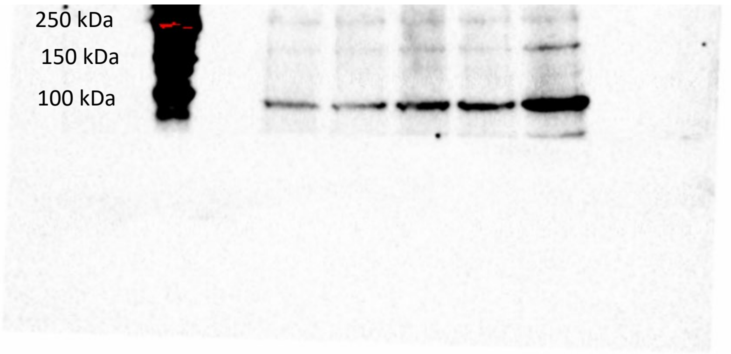

GAPDH

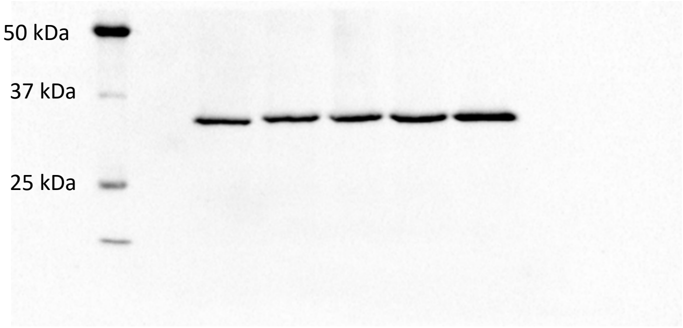

Figure 3:

Collagen 1

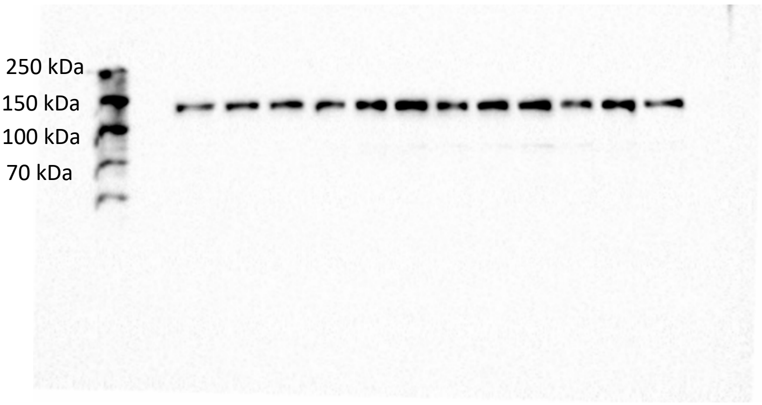

Collagen 3

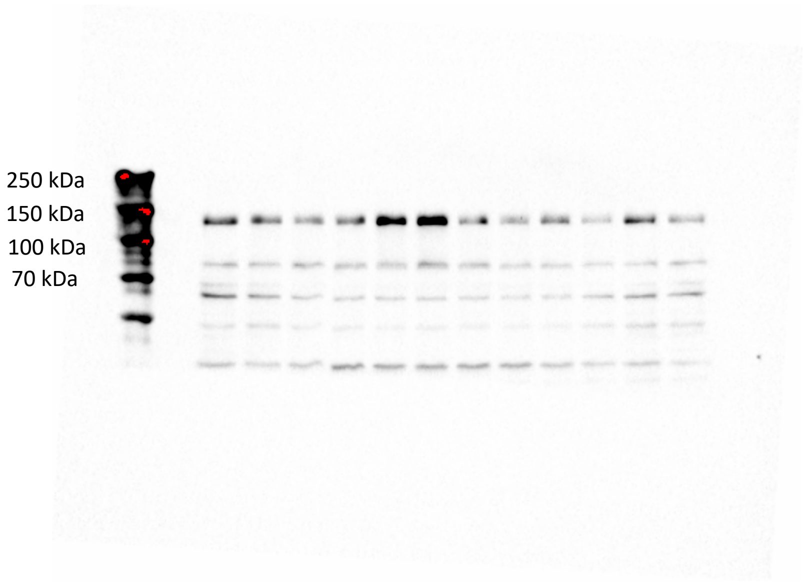

α-SMA

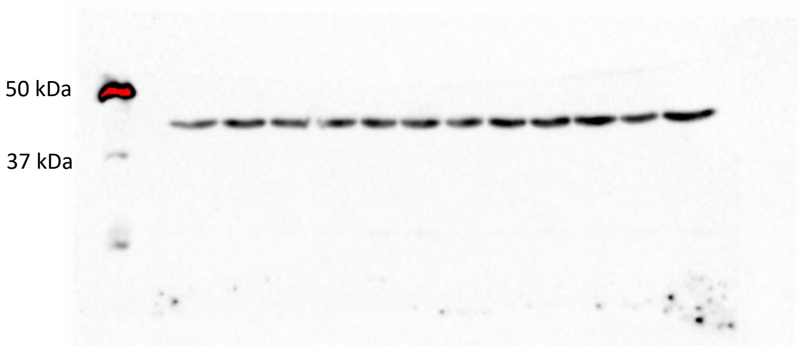

GAPDH

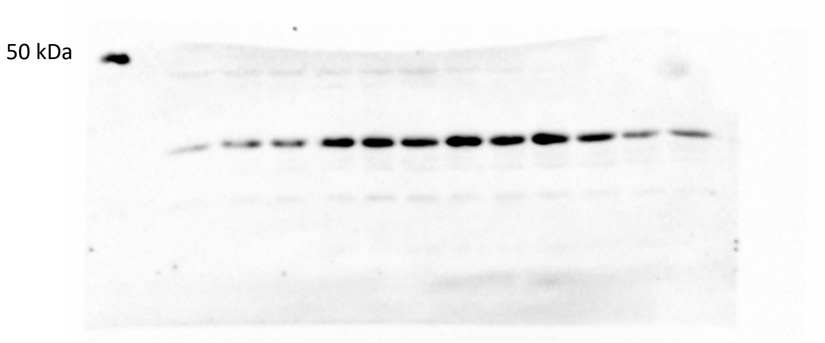

Figure 5A:

pSMAD2

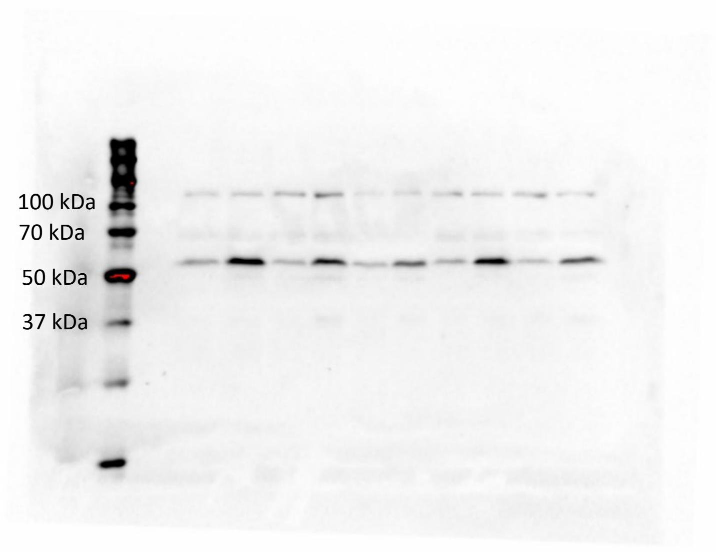

SMAD2

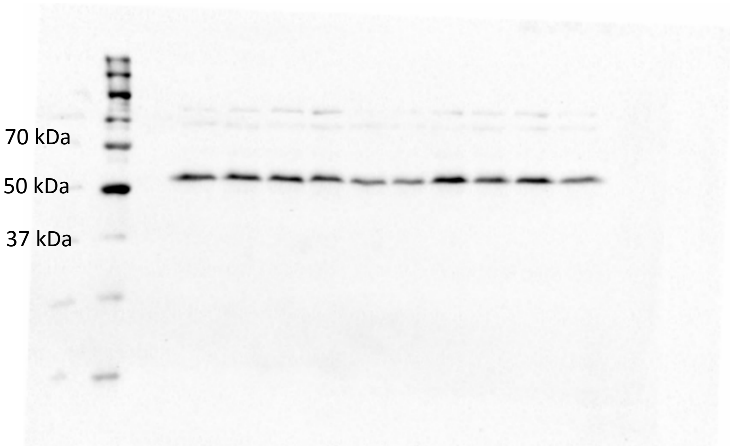

pSMAD3

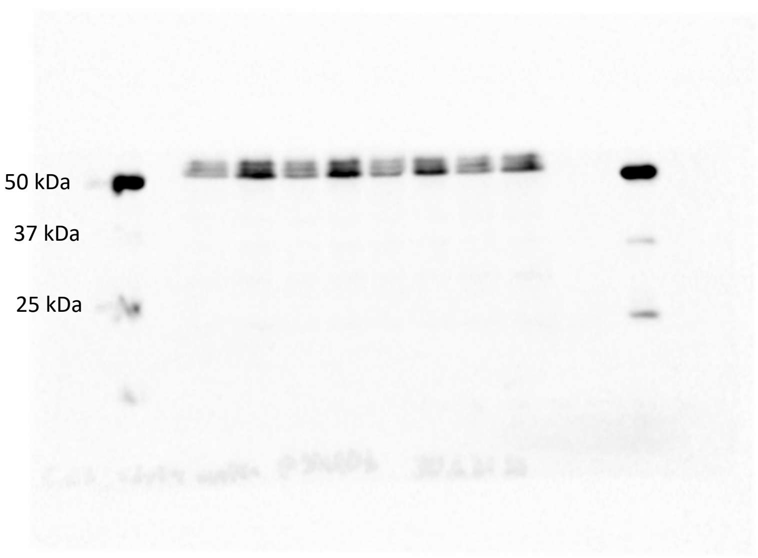

SMAD3

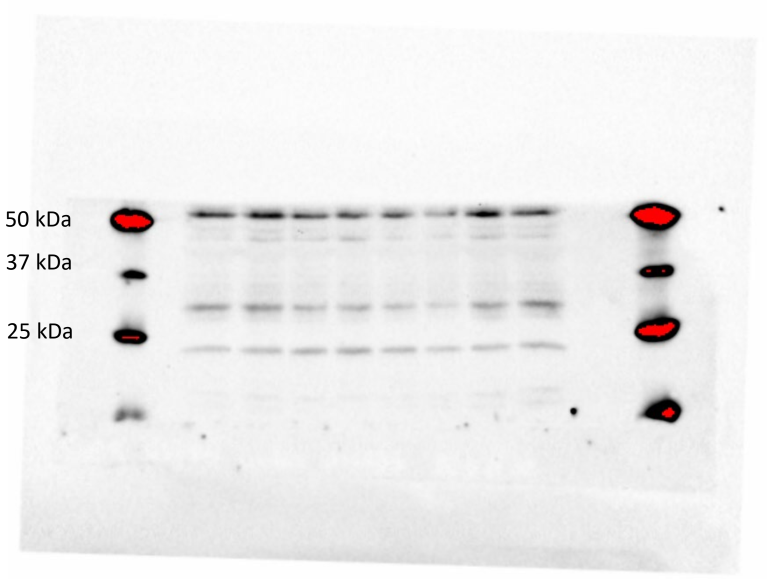

pSTAT3

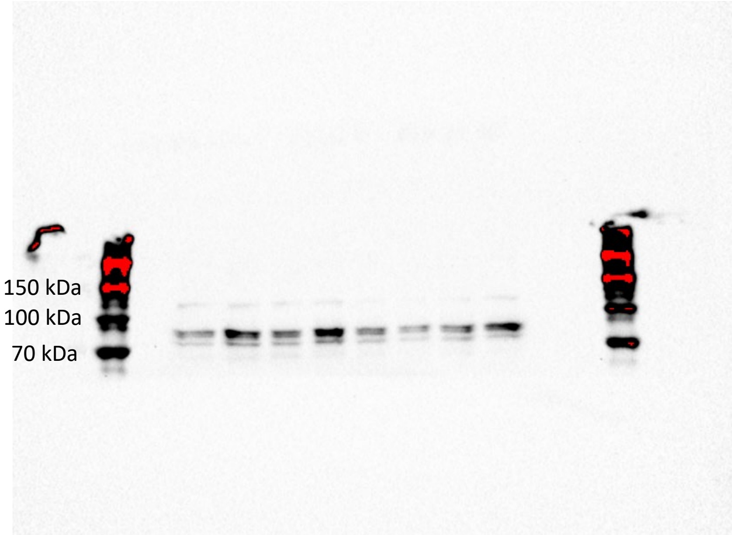

STAT3

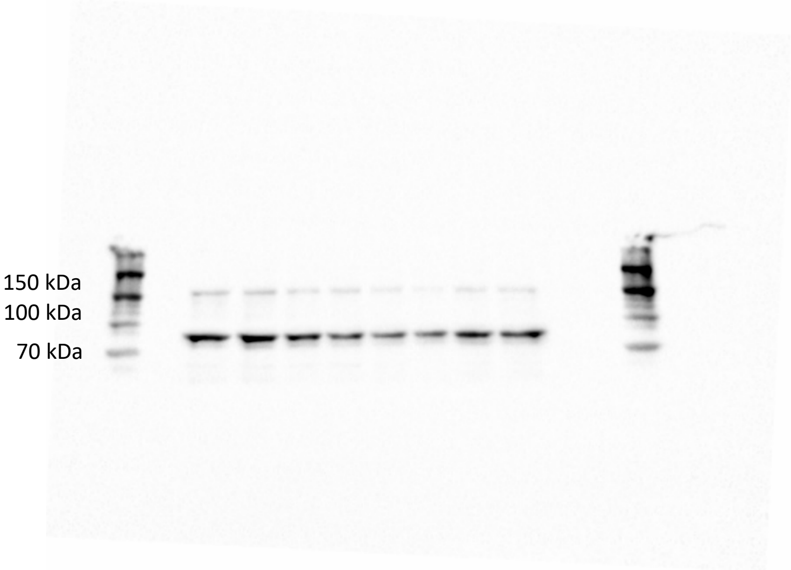

pAkt

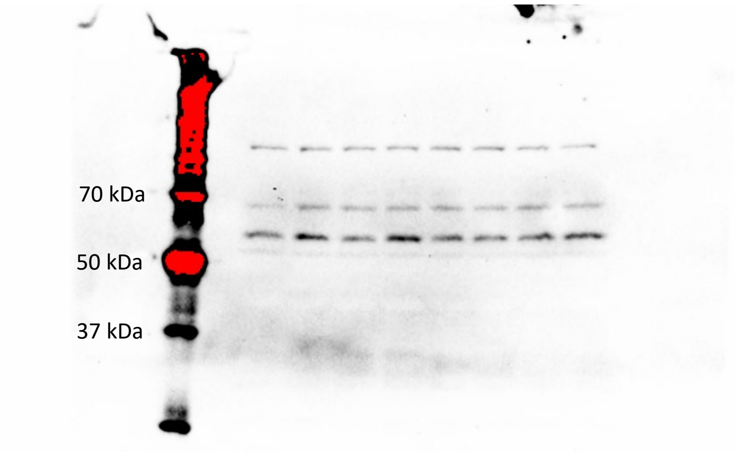

Akt

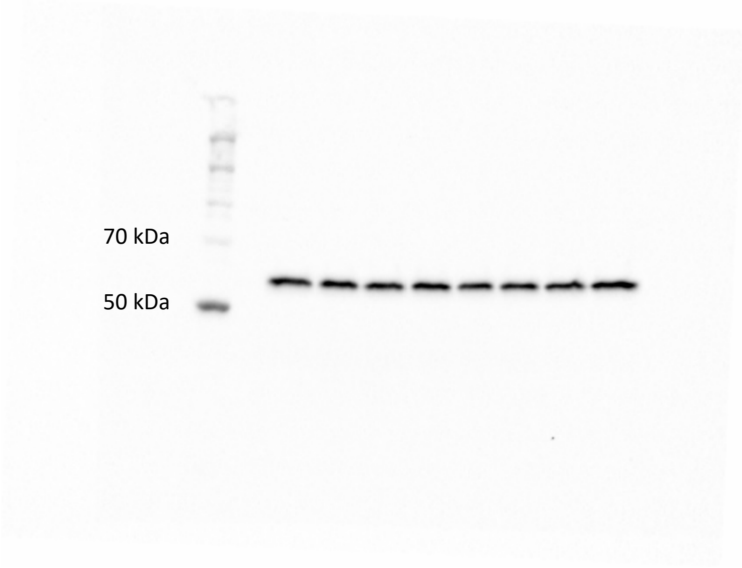

β-Actin

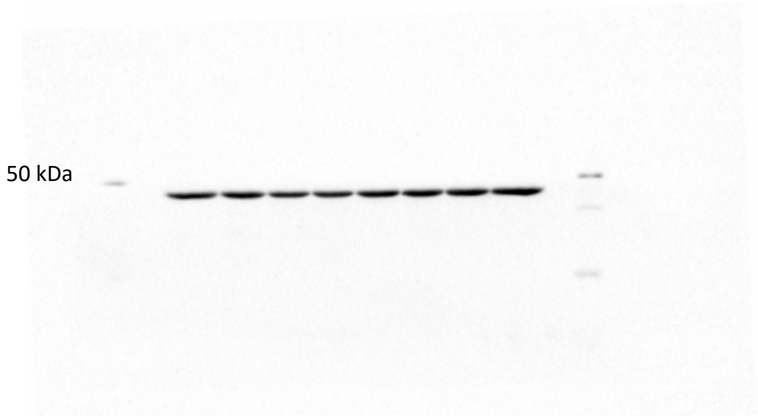

Figure 5B:

pAkt

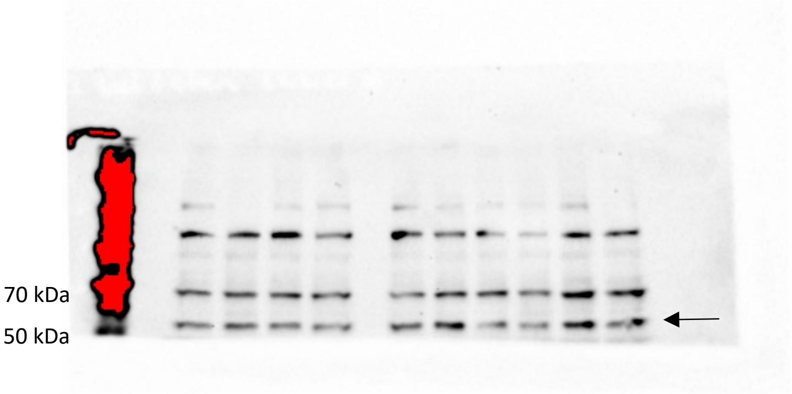

Akt

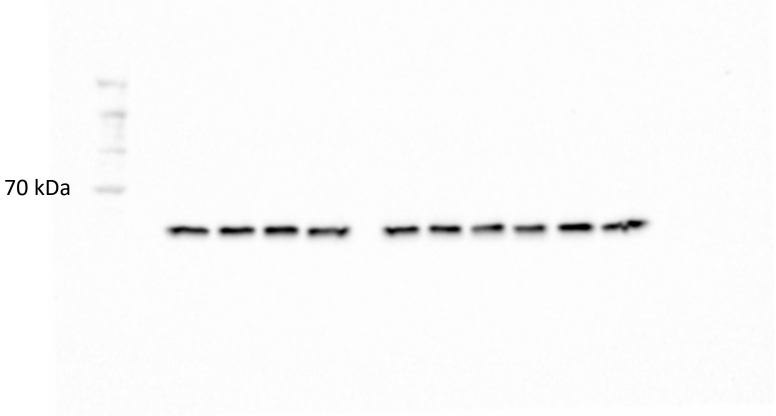

Figure 5C:

pGSK-3β

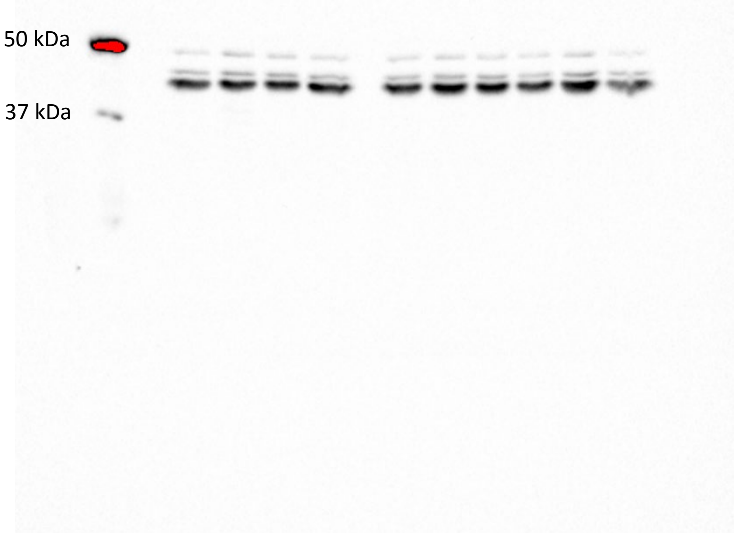

GSK-3β

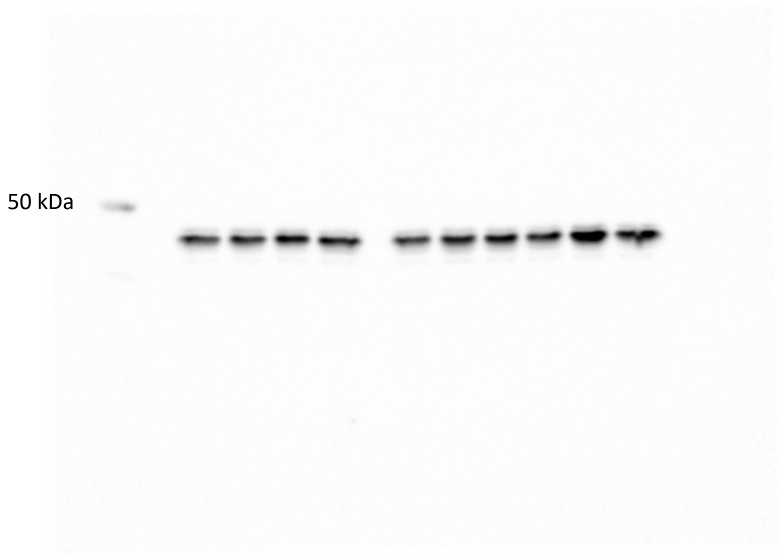

Figure 5D:

pGSK-3

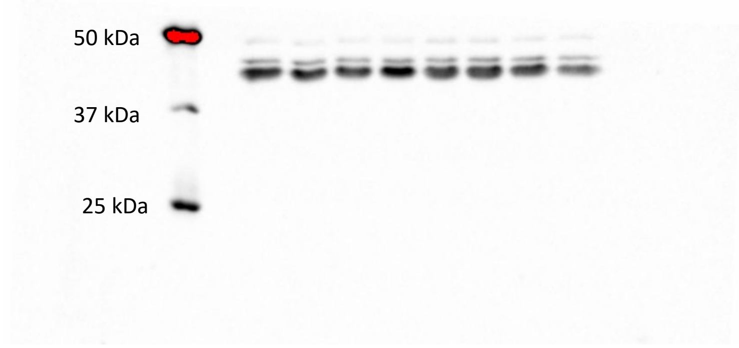

GSK-3β

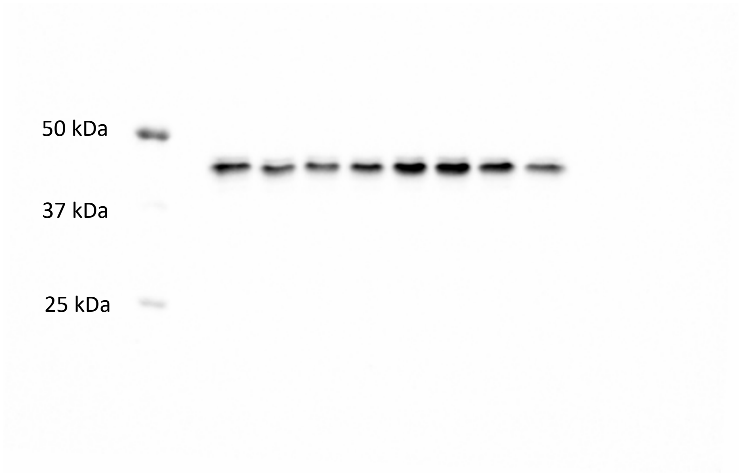

Figure 6:

Collagen I

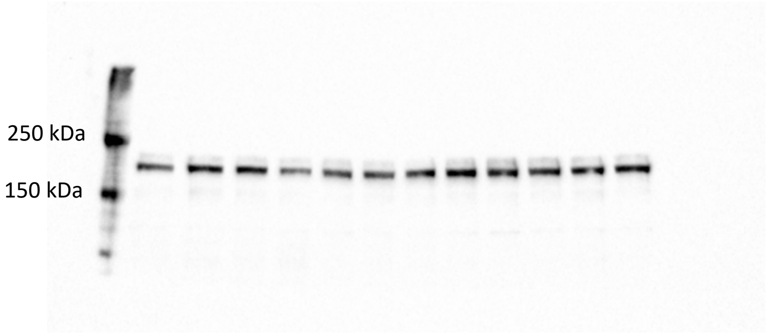

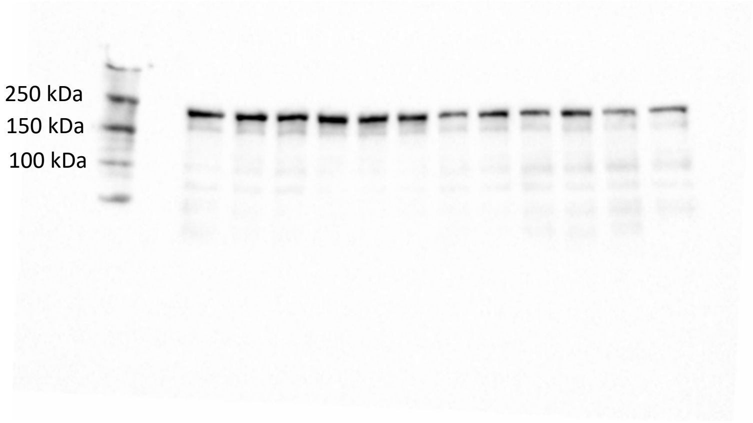

α-SMA

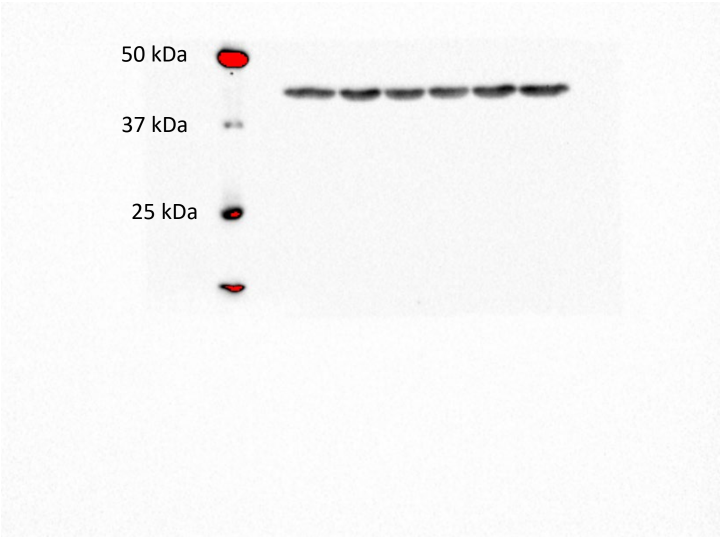

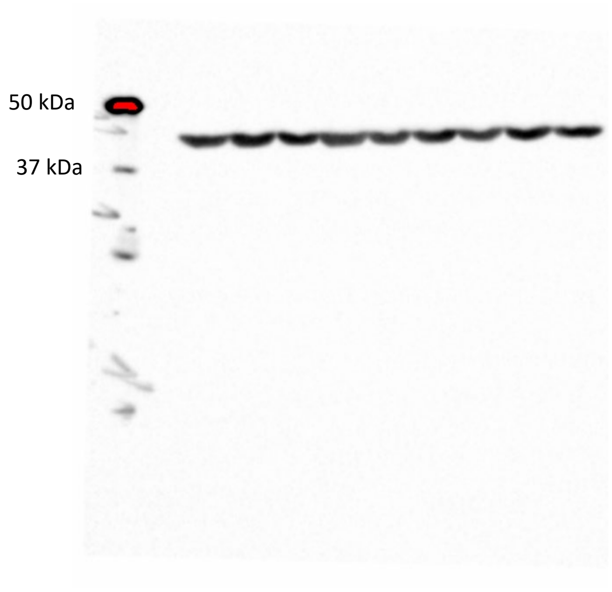

GAPDH

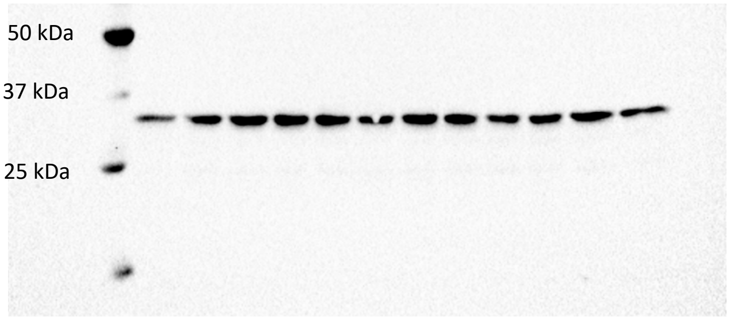

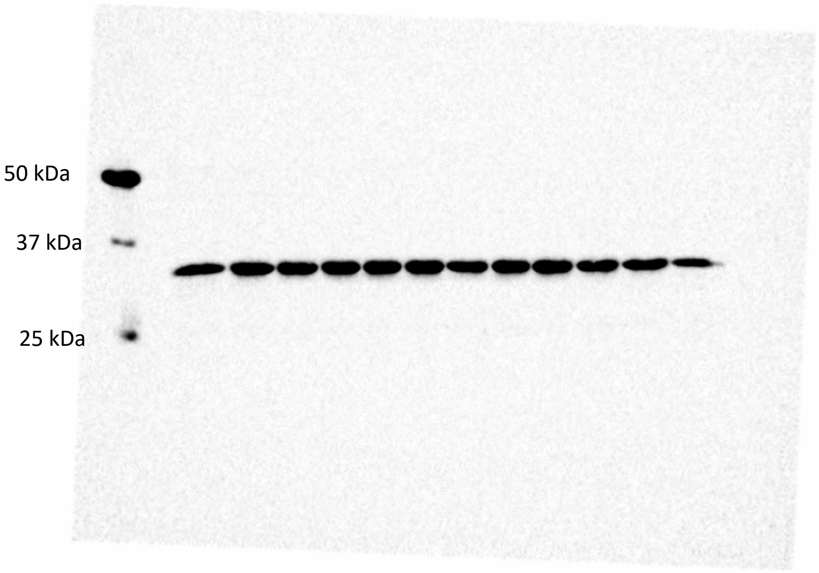

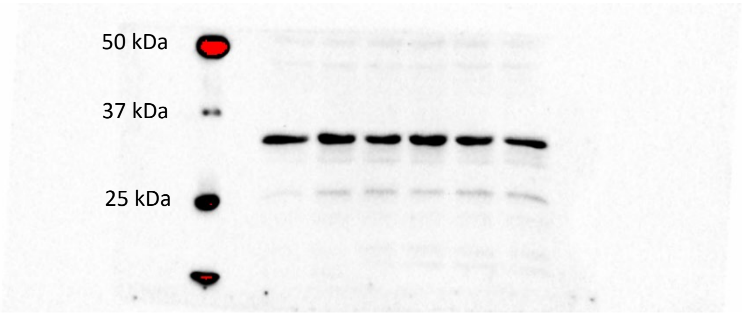
50 kDa
37 kDa
25 kDa

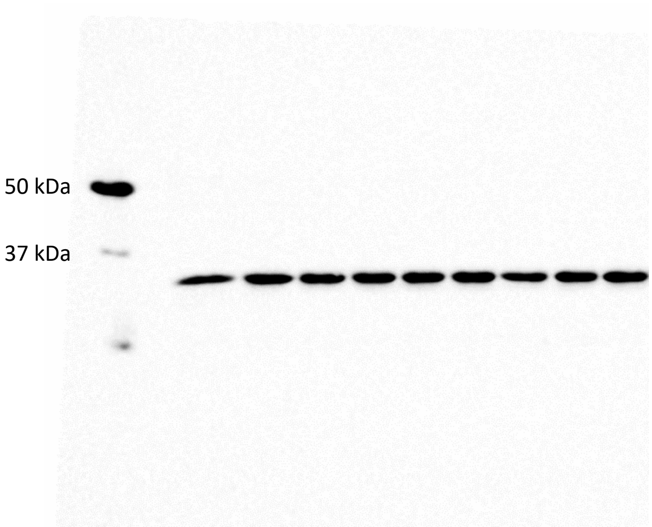
50 kDa
37 kDa
