# Supplementary material for: Impaired non‐canonical transforming growth factor‐β signalling prevents profibrotic phenotypes in cultured peptidylarginine deiminase 4‐deficient murine cardiac fibroblasts
Source: J Cell Mol Med. 2021 Sep 14;25(20):9674–84. doi: 10.1111/jcmm.16915 (PMC8505821; doi:10.1111/jcmm.16915)
Supplement: Supplementary file 2 — Fig S2 [file JCMM-25-9674-s003.pdf]

Impaired non-canonical TGF-β signaling prevents profibrotic phenotypes in cultured PAD4-deficient murine cardiac fibroblasts

Hanane Akboua[1], Kaveh Eghbalzadeh[1], Ugur Keser[1], Thorsten Wahlers[1], Adnana Paunel-Görgülü[1*]

[1]Department of Cardiothoracic Surgery, Heart Center of the University of Cologne, Cologne, Germany

Supplemental Figure 2:

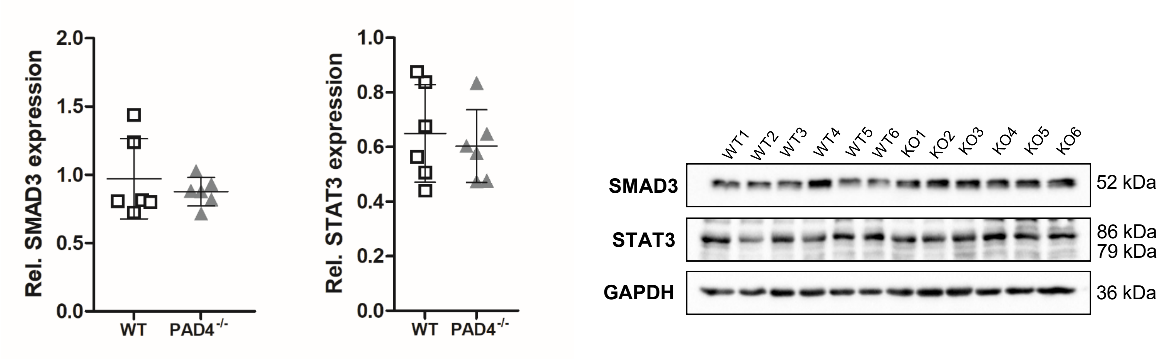

Figure S2: Cardiac expression of SMAD3 and STAT3 at baseline. The expression of SMAD3 and STAT3 was investigated in heart tissue lysates from WT (n = 6) and PAD4$^{-/-}$ (n = 6) mice by western blot. Protein expression was normalized to GAPDH levels. Protein levels were similar between mice of different genotypes.
