# Supplementary material for: Impaired non‐canonical transforming growth factor‐β signalling prevents profibrotic phenotypes in cultured peptidylarginine deiminase 4‐deficient murine cardiac fibroblasts
Source: J Cell Mol Med. 2021 Sep 14;25(20):9674–84. doi: 10.1111/jcmm.16915 (PMC8505821; doi:10.1111/jcmm.16915)
Supplement: Supplementary file 3 — Fig S3 [file JCMM-25-9674-s001.pdf]

Impaired non-canonical TGF-β signaling prevents profibrotic phenotypes in cultured PAD4-deficient murine cardiac fibroblasts

Hanane Akboua[1], Kaveh Eghbalzadeh[1], Ugur Keser[1], Thorsten Wahlers[1], Adnana Paunel-Görgülü[1*]

[1]Department of Cardiothoracic Surgery, Heart Center of the University of Cologne, Cologne, Germany

Supplemental Figure 3:

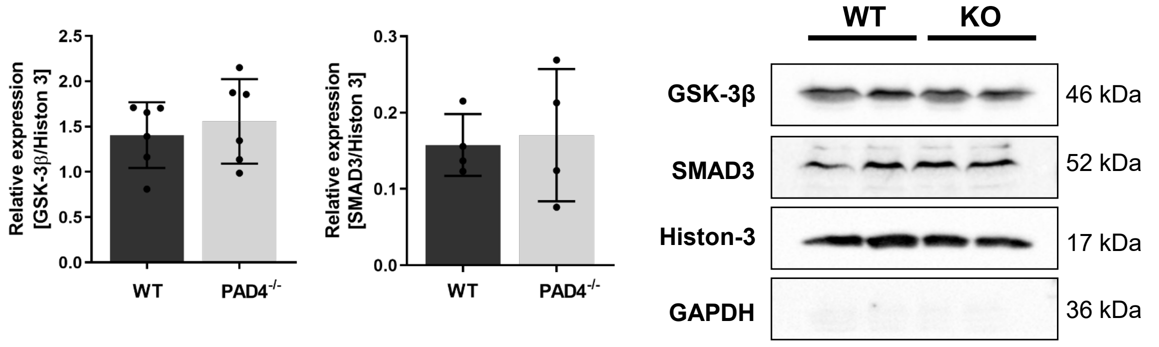

Figure S3: Nuclear localization of GSK-3β and SMAD3 in WT and PAD4$^{-/-}$ CFs. WT and and PAD4$^{-/-}$ CFs (P3) were cultured until reaching 90% confluence. After preparation of nuclear extracts, GSK-3β and SMAD3 were detected by Western blotting. Histon-3 was used for normalization. No differences between WT and PAD4$^{-/-}$ CFs were detected. Representative western blots are depicted. n = 4-6 / group.
